# Prenatal diagnosis of Meier-Gorlin syndrome 7: a case presentation

**DOI:** 10.1186/s12884-021-03868-5

**Published:** 2021-05-17

**Authors:** Xia Li, Lan-Zhen Zhang, Lin Yu, Zhao-Lua Long, An-Yun Lin, Chen-Yu Gou

**Affiliations:** 1 Department of Obstetrics, the Second Affiliated Hospital of Guangzhou Medical University, No. 250 Changgang East Road, Haizhu District, Guangzhou, 510260 Guangdong Province China; 2 Department of Radiology, the Second Affiliated Hospital of Guangzhou Medical University, Guangzhou, Guangdong Province China; 3 Fetal Medicine Center, Department of Obstetrics and Gynecology, Sixth Affiliated Hospital of Sun Yat-Sen University, No. 26 Yuancun Erheng Road, Guangzhou, 510655 China

**Keywords:** Meier-Gorlin syndrome 7 (MGS7), Intrauterine growth restriction, CDC45, Compound heterozygous mutations, Whole-exome sequencing (WES)

## Abstract

**Background:**

Meier-Gorlin syndrome 7 (MGS7) is a rare autosomal recessive condition. We reported a fetus diagnosed with Meier-Gorlin syndrome 7. The antenatal sonographic images were presented, and compound heterozygous mutations of CDC45 on chromosome 22 were identified by whole-exome sequencing (WES).

**Case presentation:**

Fetal growth restriction (FGR), craniosynostosis, and brachydactyly of right thumb were found in a fetus of 28th gestational weeks. The fetus was diagnosed as MGS7 clinically. After extensive counseling, the couple opted for prenatal diagnosis by cordocentesis and termination of pregnancy. Karyotype analysis and WES were performed. Chromosomal karyotyping showed that the fetus was 46, XY. There were 2 mutations of CDC45, the causal gene of MGS7 on chromosome 22, which were inherited from the couple respectively were identified by WES. Facial dysmorphism, brachydactyly of right thumb, and genitalia abnormally were proved by postpartum autopsy, and craniosynostosis was confirmed by three-dimensional computed tomography (3D-CT) reconstruction.

**Conclusions:**

It is possible to detect multiple clinical features of Meier-Gorlin syndrome in prenatal sonography. Deteriorative FGR complicated with craniosynostosis indicates MGS7. Combination of 2D and 3D ultrasonography helps to detect craniosynostosis. The affected fetus was confirmed a compound heterozygote of CDC45 related MGS by whole-exome sequencing, which is critical in identifying rare genetic diseases.

## Background

Meier-Gorlin syndrome 7 (MGS7) (MIM; 224,690) is a rare autosomal recessive condition, approximately 1–9 per million births according to the literature [1]. Yet, the morbidity of MGS might be underestimated due to insufficient recognition, under-reporting, or missed diagnoses. Severe intrauterine and postnatal growth retardation, microtia, and patellae aplasia/hypoplasia are typical clinical characteristics of MGS7 [2], and the associated congenital malformations may range from microcephaly, congenital pulmonary emphysema, urogenital anomalies to skeletal abnormalities [1]. MGS is often characterized by specific facial features [1, 3]. Eight types of MGS were classified according to the slightly different phenotypes [4]. It is reported recently that allelic mutations in the CDC45 cause MGS7 [2].

## Case report

A 23-year-old natural-conceived Chinese woman, gravida 1, para 0; underwent four ultrasound examinations in the local hospital. The first sonography was performed at 52 days post menstruation. It revealed the crown-rump length (CRL) of the embryo was 4 mm, which was consistent with 6 + 2 weeks of the gestational age based on the ultrasound estimated stander of the ethnic Chinese population [5].

**Fig. 1:**
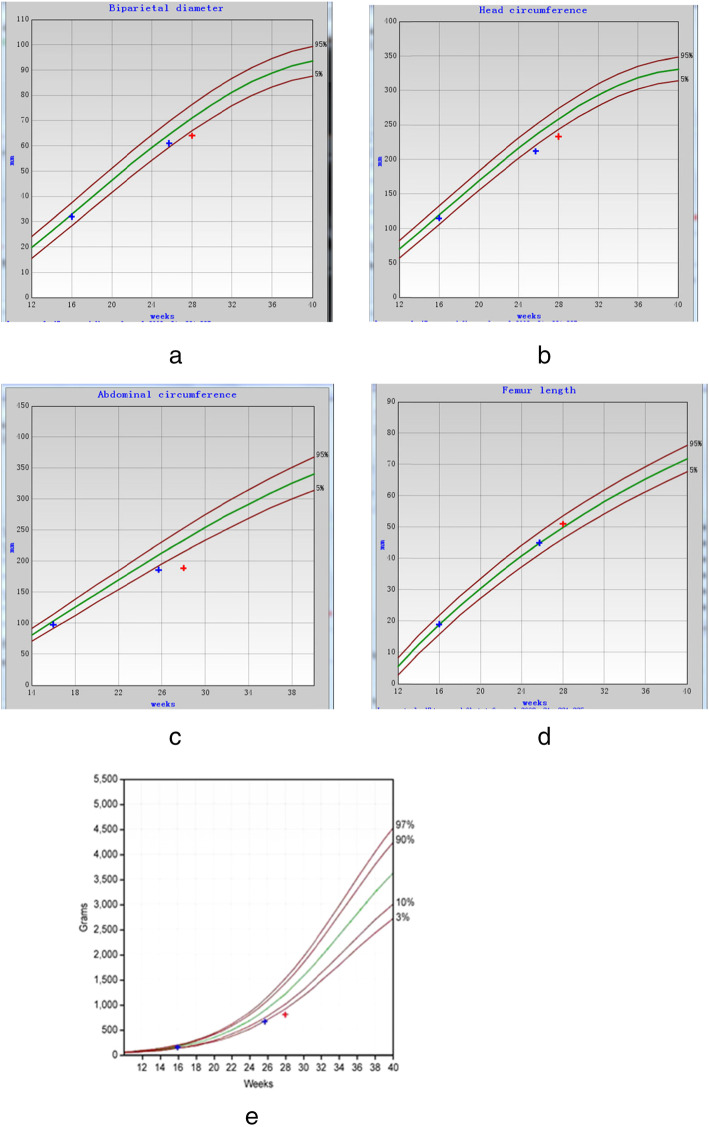
Fetal biometric parameters by ultrasound monitoring. **a** Biparietal diameter (BPD) **b** Head circumference (HC) **c** Abdominal circumference (AC). **d** Femur length (FL) **e** Estimated Fetal Weight (EFW). Progressive fetal growth retardation could be described by the growth curve. The Standard curve was plotted based on data of ethnic Chinese population [4, 5]

At 11 + 5 weeks, sonography showed the fetal CRL was 47 mm, which was in the 68th percentile of the Chinese population. Two more ultrasounds were performed at 16 + 0 weeks and 25 + 5 weeks. FGR was diagnosed, and microcephalus was suspected. (Fetal biometric parameters were showed in Table 1 and Fig. 1).

**Table 1:** The fetal intrauterine parameters

	BPD (mm)	Percentile (SD)	HC (mm)	Percentile (SD)	AC (mm)	Percentile (SD)	FL (mm)	Percentile (SD)	EFW (g)	Percentile (SD)
16^+0^W	32	35.7% (− 0.37)	115	28.9% (− 0.56)	98	22.7% (− 0.75)	18	31.9% (− 0.47)	129	18.9% (− 1.45)
25^+5^W	61	12.9% (−1.14)	212	0.5% (−2.58)	186	1.3% (−2.23)	45	52.1% (0.04)	650	2.1% (−2.8)
28^+0^W	64	1.3% (−2.24)	233	0.3% (−2.78)	189	under0.1% (−3.85)	51	67.3% (0.45)	792	under0.1% (−3.89)

The patient was referred to our hospital at 28 weeks of gestational age. Ultrasound scan at 28 weeks revealed that deterioration of fetal growth retardation: fetal estimated weight was in 0.3 percentile, biparietal diameter was in 1.3 percentile (− 2.24 SD), head circumference was in 0.3 percentile (− 2.78 SD), abdominal circumference was under 0.1 percentile (− 3.85 SD) [6].

**Fig. 2:**
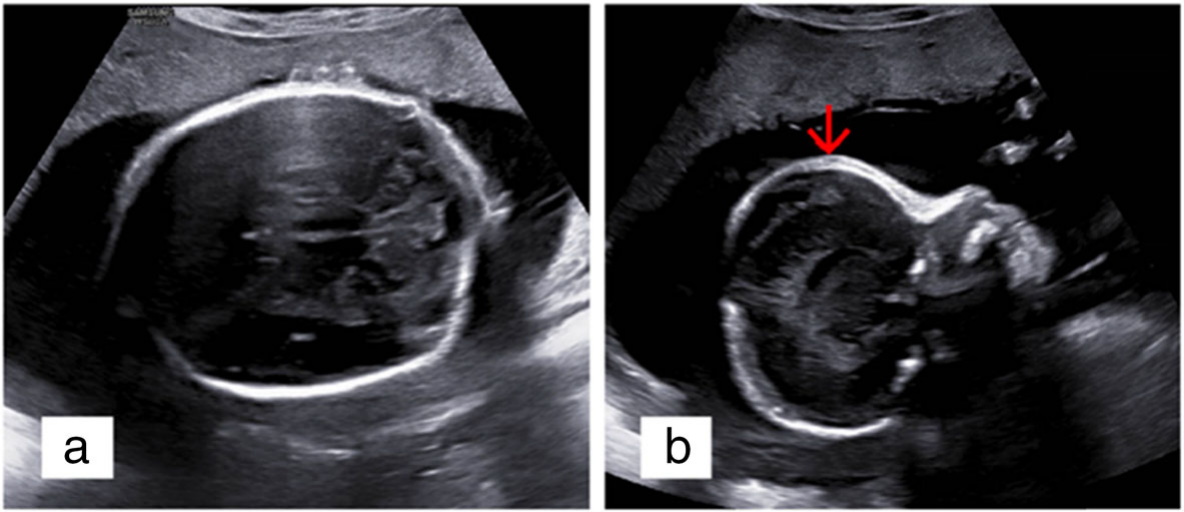
Indirect signs of craniosynostosis showed by 2D prenatal ultrasound scanning. **a** brachycephaly with an 77% CI, absence of lucency at the coronal suture, and the brain shadowing sign were showed in fetal cross-section. **b** Turribrachycephaly was showed in sagittal section. Red arrow: frontal bossing

**Fig. 3:**
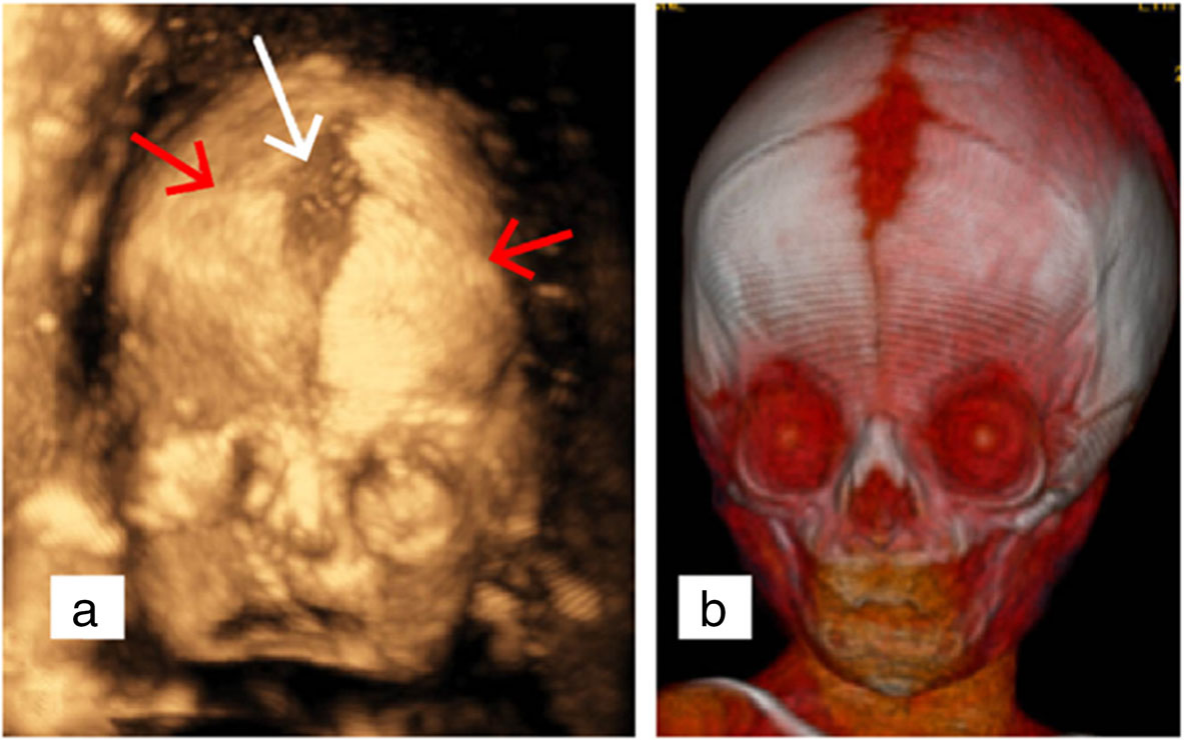
Bilateral coronal synostosis showed by 3D ultrasonography and 3D CT. **a** A shortened and broader skull in the antero-posterior dimension (brachycephaly), extremely narrow coronal sutures (red arrow) and deformed anterior fontanelle (white arrow) were by prenatal 3D ultrasound using surface-rendered pattern in the skeletal mode. **b** Postpartum 3D CT reconstruction of skull showed typical characteristics of bilateral coronal synostosis, which were almost exactly the same as 3D ultrasound

**Fig. 4:**
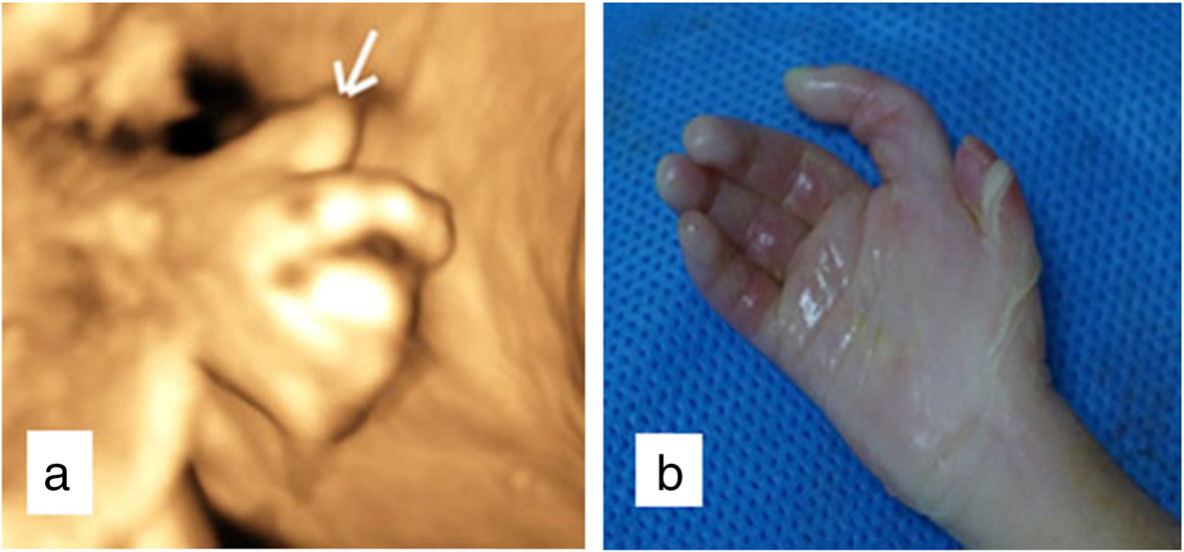
Brachydactyly of the right thumb. **a** prenatal 3D ultrasound **b** postpartum autopsy

In addition to FGR, an abnormal cranial shape was observed. A shortened and broader skull in the anteroposterior dimension (brachycephaly), frontal bossing in sagittal section (turribrachycephaly), absence of lucency at the coronal suture, and the brain shadowing sign in the cross-section was showed in two-dimensional (2D) ultrasound screening. These sonographic findings were bilateral coronal synostosis characteristics, and fetal coronal synostosis was confirmed by skeletal mode of 3D ultrasonography (Figs. 2 and 3). Brachydactyly of right thumb was also detected (Fig. 4), but no positive findings were presented in fetal echocardiography. Based on sonographic features, Meier-Gorlin syndrome and Pfeiffer syndrome (MIM: 101600) were suspected.

After detailed counseling, the couple opted for prenatal diagnosis by cordocentesis and pregnancy termination regarding the diagnosis and prognosis, despite expectations for an average life span and intellect. The couple gave written consent for termination, karyotyping with cord-blood, pedigree analysis of WES with fetal tissue, and autopsy, but they refused chromosome microarray analysis (CMA). The hospital Ethics Committee approved all the procedures.

**Fig. 5:**
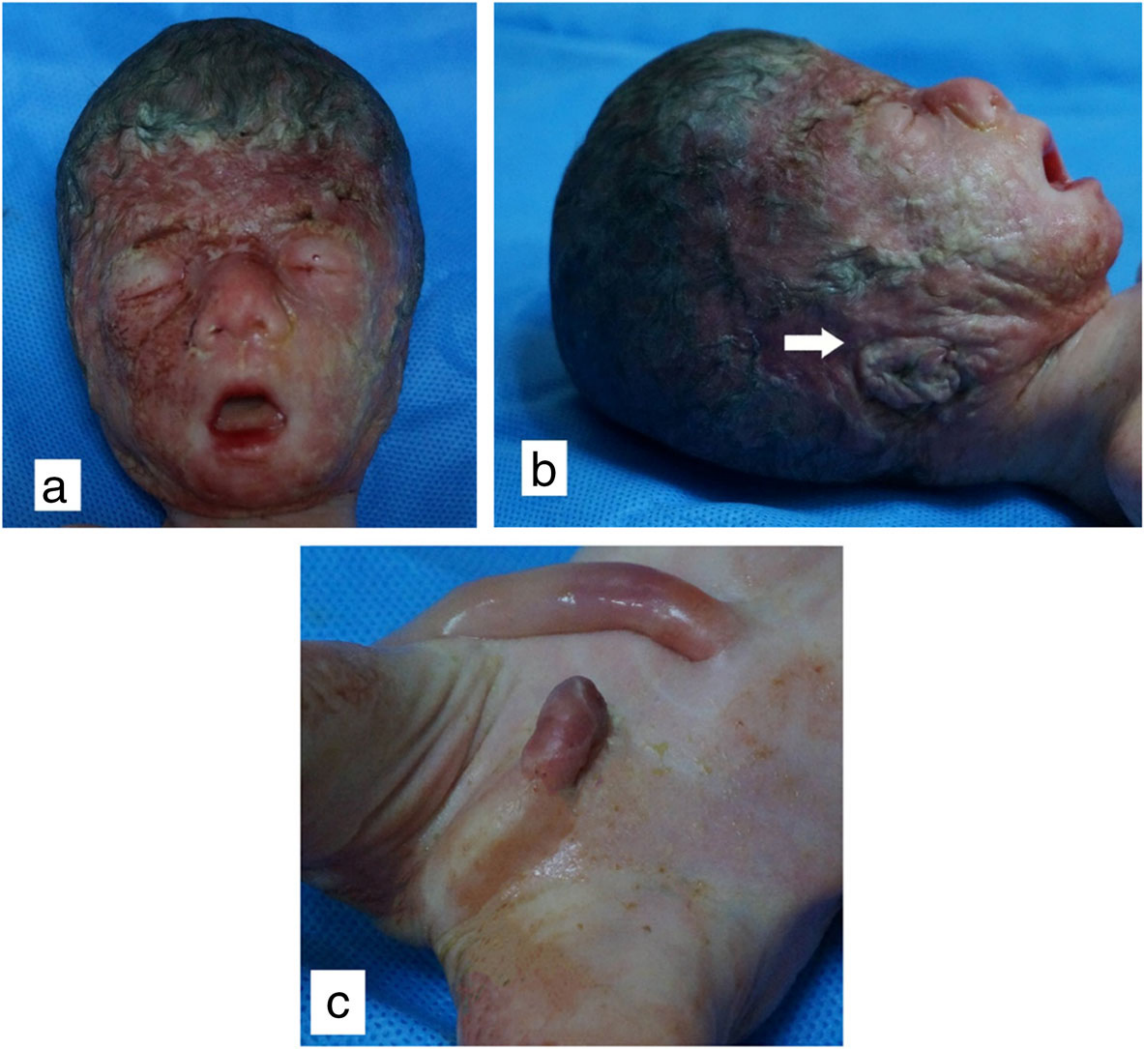
Postpartum autopsy findings of the affected fetus. **a** A shortened and broader skull in the antero-posterior dimension (brachycephaly). **b** Turribrachycephaly and microtia (white arrow) were shown in sagittal section. **c** Anomalies of the urogenital tract, cryptorchidism

**Fig. 6:**
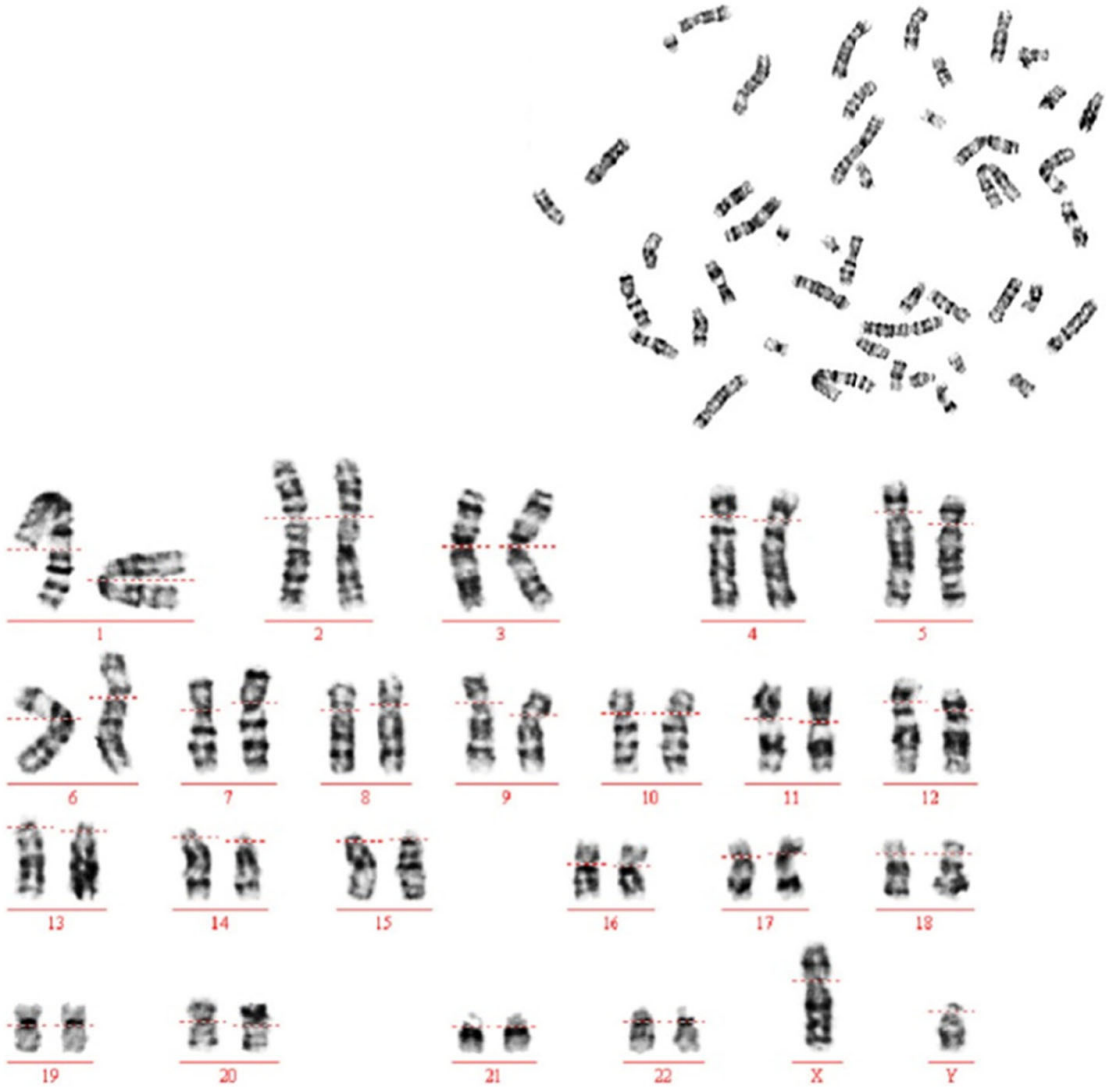
chromosome karyotype of the affected fetus (46,XY)

The aborted male fetus, with Scrotal dysplasia (Fig. 5), weighed 710 g (under 0.1% percentile), with a length of 40 cm. Typical MGS facial features (Fig. 5), including proptosis, microtia, microstomia, prominent nose, and a convex profile, were showed at fetal autopsy postpartum. Brachydactyly of right thumb was consistent with the previous sonographic finding. Bilateral coronal synostosis confirmed by 3D-CT reconstruction postpartum. MGS could be diagnosed clinically. Chromosomal karyotyping showed that the fetus was 46, XY (Fig. 6), no chromosome aberration in the fetus was found.

**Fig. 7:**
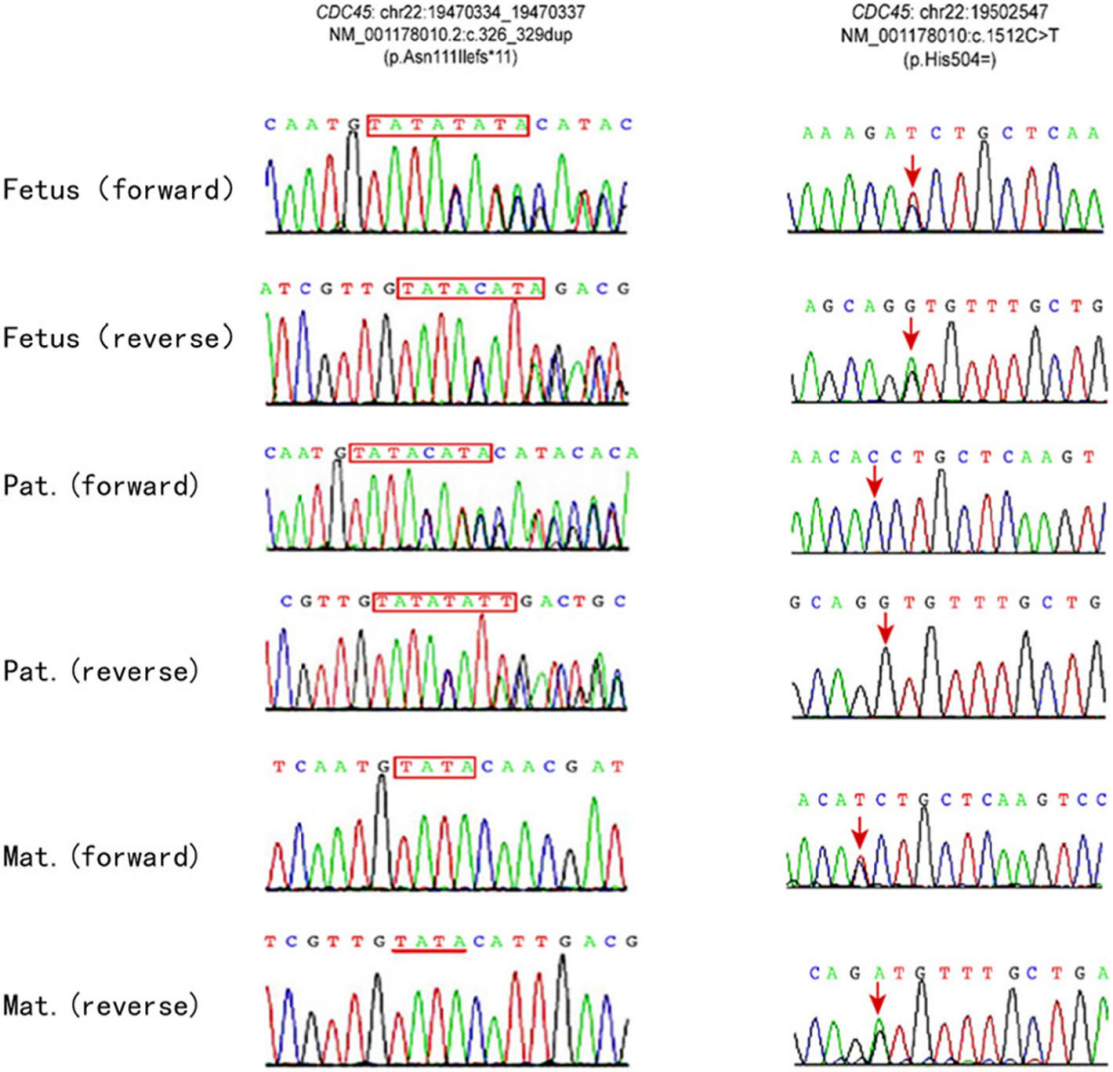
compound heterozygous mutations of CDC45 inherited from parents. (by Sanger sequencing). CDC45c.326_329dup (p.Asn111Ilefs*11) is a frameshift mutation and paternal inherited. c.1512C > T (p.His504=) is a is a site-directed mutation and maternal inherited

DNA was extracted from fetal tissue and the couple’s peripheral blood. WES by targeted-capture and high throughput sequencing and pedigree analysis was performed. WES pedigree analysis revealed compound heterozygous mutations of CDC45 on chromosome 22 in the fetus, which were inherited from the couple, respectively. CDC45 is regarded as the causal gene of MGS7 [MIM: 617063], an autosomal recessive disease. The paternal inherited mutation NM_001178010.2: c.326_329dup (p.Asn111Ilefs*11) (chr22: 19470334_19470337) (Fig. 7) is pathogenetic because it causes a reading frame shift and probably leads to premature termination of protein-coding sequence (protein length change, PM4). The maternal inherited mutation NM_001178010.2: c.1512C > T (p.His504 =) (Fig. 7) is a site-directed mutation. It is evaluated as a variation of uncertain significance because it is a synonymous mutation. These two mutations and their genetic source were confirmed by Sanger sequencing as well.

## Discussion and conclusions

MGS, which has also been called the ear-patella-short stature syndrome, is a part of the primordial dwarfism spectrum [1, 7]. In previous eras, MGS was diagnosed clinically based on the classical triad of microtia, absent or hypoplastic patellae, and short stature after birth [8]. MGS appears to present with different features; approximately 18% of patients present only two of three clinical features [9]. Advances in ultrasound screening and application of 3D ultrasonography conduce to prenatal diagnosis of MGS. Classification of MGS depends on the genetic diagnosis. ORC1, ORC4, ORC6, CDT1, CDC6, GMNN, CDC45, MCM5 have been reported in MGS1 to MGS8, respectively.

FGR presents in 97% of MGS patients [8, 10–14] and is deteriorative, and it might be early onset. However, the literature on intrauterine growth data of MGS fetuses is limited. Microcephaly is a noticeable feature of MGS in prenatal ultrasound screening, while microtia can be easily missed, thought microtia present in most MGS cases. Congenital cardiac anomalies are also reported in approximately 7–10% of MGS patients; fetal echocardiography can be an approach to apply. But other clinical manifestations such as skeletal anomalies (absent patellae, scoliosis, and syndactyly), genital anomalies (cryptorchidism, micro-penis, hypoplasia of the corpora cavernosa hypospadias), respiratory malformation (pulmonary emphysema, laryngomalacia, tracheomalacia, and bronchomalacia) [1, 4, 7, 10, 14, 15] were hard to detect in prenatal ultrasound screening. In our case, we identified brachydactyly of the right thumb as evidence of skeletal anomaly prenatally, yet microtia and genital abnormalities were found postpartum.

Craniosynostosis, the process of premature fusion of one or more of the cranial sutures, is reported as a characteristic to differentiate MGS7 from the other types of MGS [7]. Yet, there are relatively rare reports on prenatal ultrasound examination of cranial sutures by 2D scanning and 3D reconstruction, and the detection rate of craniosynostosis is low [16, 17]. It was challenging to diagnose craniosynostosis prenatally because cranial sutures could not be visualized directly in 2D ultrasound scanning. Still, indirect signs such as abnormal cephalic index (CI), cranial shape, notch at the level of coronal sutures, or thickening of the calvarium in the suture region should prompt the diagnosis. The CI is a proportion of biparietal diameter (BPD) to occipitofrontal diameter (OFD), which gives an idea of the fetal head shape. CI is considered normal from 75 to 85%. Dolichocephaly is defined by a CI below 75% and brachycephaly by a CI above 85%. In recent years, coronal sutures can be measured using skeletal and surface rendering modes of 3D ultrasonography [18]. Although 3D-CT is a valuable diagnostic modality, radiation exposure of survival fetus must be considered [19].

But craniosynostosis is involved in a wide range of syndromes, such as Pfeiffer, Apert, Crouzon, Shaethre-Chotzen, and Muenke syndromes. Each syndrome has some special features to differentiate from the others, for example, brachydactyly in Pfeiffer syndrome, broad metopic suture, and absent coronal sutures in Apert syndrome. Therefore, it is extremely relevant to detail fetal anatomy as a whole. Particular attention should be paid to fetal hands and feet, long bone growth, central nervous system, and heart [3]. 3D ultrasound has the advantage of providing additional information.

In our presented case, craniosynostosis was diagnosed when shortened, and broader skull in the anteroposterior dimension, frontal bossing in sagittal section, and extremely narrow coronal sutures showed by 2D/3D ultrasonography. We preferred the diagnosis of GMS7 because of the deteriorative FGR, yet the possibility of Pfeiffer syndrome could not be ruled out considering brachydactyly of right thumb. Finally, it was confirmed CDC45 related GMS7 by WES. Therefore, genetic diagnosis is a critical strategy for identifying rare genetic disorders.

It is reported that biallelic mutations cause MGS7 in CDC45 [12]. CDC45 is a critical helicase activator essential for DNA replication initiation and elongation [13]. The protein is involved in pre-initiation complex formation, which initiates DNA replication origin firing and DNA synthesis in S-phase cycles [8]. Impaired pre-replication complexes cause a reduction of cell proliferation, which might be the pathogenesis of FGR and craniosynostosis.

WES results of the presented case identified compound heterozygous mutations of CDC45 in the fetus. CDC45(c.326_329dup [p.Asn111Ilefs*11]), paternal inherited mutation, is at exon 1, the duplicated bases cause reading frame shift and lead to premature termination of protein-coding sequence (protein length change, PM4). Maternal inherited mutation c.1512C > T (p.His504 =) is a synonymous mutation at exon 14, which shall not result in a change to amino acid sequence in theory. But clinical symptoms of the affected fetus was consistent with MGS7, an autosomal recessive disease, and no other pathogenetic mutations were found. The case seemed to be not in accordance with Mendel’ s law of inheritance. CDC45(c.1512C > T [p.His504 =]) is close to initial segment of exon 14, which might results in a malfunctional protein or a non-functional isoform by altering splicing of physiological CDC45 transcripts, though it is a synonymous mutation. More research should be done to evaluate pathogenicity of this mutation. According to the guidelines of fetal exome sequencing in prenatal diagnosis, the fetus presented coincident clinical features with MGS7 caused by CDC45 mutations, c.1512C > T (p.His504 =) should be reported and could be evaluated as a variation of uncertain significant [20–22].

One defect of this study was that CDC45 transcripts of the affected fetus were not verified by Reverse Transcription-polymerase chain reaction (RT-PCR). The other inadequacy was the absence of CMA data. An alternative also should be considered, although less likely, dysfunction of maternal inherited CDC45 might be caused by copy number variation (CNVs), not the c.1512C > T mutation.

WES often plays a conclusive role in identifying rare genetic diseases, some of which might present similar manifestations or stay undiagnosed by conventional approaches, such as single-gene testing or panel testing, or CMA. WES also has excellent advantages in the differential diagnosis of genetically heterogeneous disorders with atypical or incomplete clinical features, just as in the GMS case we reported.

In summary, deteriorative FGR, shortened and broader skull in the anteroposterior dimension, frontal bossing in sagittal section showed in 2D screening, and highly narrow coronal sutures showed by 3D ultrasonography shall remind sonographer of MGS7 and to search more implications. Compound heterozygous mutations of CDC45 identified by WES provide reference to genetic counseling and fetal diagnosis for subsequent pregnancy.

It is possible to detect multiple clinical features of Meier-Gorlin syndrome in prenatal sonography. Deteriorative FGR complicated with craniosynostosis indicates MGS7. Combination of 2D and 3D ultrasonography helps to detect craniosynostosis. The affected fetus was confirmed a compound heterozygote of CDC45 related MGS by whole-exome sequencing, which is critical in identifying rare genetic diseases.

## Data Availability

Datasets from this study are available upon reasonable request from the corresponding author.

## Abbreviations

MGS 7: Meier-Gorlin syndrome 7
WES: Whole-exome sequencing
FGR: Fetal growth restriction
3D-CT: Three-dimensional computed tomography
CRL: Crown-rump length
CMA: Chromosome microarray analysis
VUS: Variation of Uncertain Significance
CI: Cephalic index
BPD: Biparietal diameter
OFD: Occipitofrontal diameter
RT-PCR: Reverse Transcription-polymerase chain reaction
CNVs: Copy number variation (CNVs)
